# XsymNet: Combined Exhaustive Symmetry and Machine Learning for Phase Transition Studies

**DOI:** 10.1063/4.0001037

**Published:** 2025-10-27

**Authors:** Dayton G. Kizzire, Maksim Eremenko, Matt Tucker, Yuanpeng Zhang

**Affiliations:** Oak Ridge National Laboratory, Oak Ridge, Tennessee, USA

## Abstract

Revealing the symmetry change across a phase transition is fundamentally important to understanding and controlling properties such as polarization (ferroelectric transition), conductivity (metal-insulator transition) and other unconventional properties including piezoelectricity, multiferr=ics, and superconductivity. The recently developed exhaustive symmetry search (ESS) technique has been proven to be an effective tool for systematically studying subtle and complex phase transitions. Here we present XsymNet, a combined machine learning and exhaustive symmetry search approach that has been developed to identify the subgroup symmetry of a material from powder diffraction. XsymNet is a convolutional neural network with multi-head attention trained on simulated diffraction data of members of the ISODISTORT subgroup tree. In this work we discuss the workflow of XsymNet, how it lowers the barrier for phase transition studies, and our future work towards automated diffraction analysis powered by ML.

